# Estimation of three-dimensional long axes of the maxillary and mandibular first molars with regression analysis

**DOI:** 10.1007/s12565-019-00506-1

**Published:** 2019-10-25

**Authors:** Kazuto Terada, Takashi Kameda, Ikuo Kageyama, Makoto Sakamoto

**Affiliations:** 1 Orthodontic Dentistry, The Nippon Dental University Niigata Hospital, 1-8 Hamaura-cho, Chuo-ku, Niigata, 951-8580 Japan; 2 Department of Orthodontics, The Nippon Dental University at Niigata, 1-8 Hamaura-cho, Chuo-ku, Niigata, 951-8580 Japan; 3 Department of Anatomy, The Nippon Dental University at Niigata, 1-8 Hamaura-cho, Chuo-ku, Niigata, 951-8580 Japan; 4 Department of Health Sciences, Niigata University School of Medicine, 2-746 Asahimachi-dori, Chuo-ku, Niigata, 951-8518 Japan

**Keywords:** Cone-beam computed tomography image, Least squares regression, Long axis of molar, Principal component analysis, Three-dimensional analysis

## Abstract

The purpose of this study was to determine the long axes of molars with multiple roots through ordinary least squares regression (LSR) and to compare them with the axes defined by principal component analysis (PCA). Three-dimensional radiological images of 20 dry skulls were obtained by cone-beam computed tomography (CBCT). Data from maxillary and mandibular first molars were extracted from the CBCT DICOM data with a three-dimensional image visualization system. The obtained data were reconstructed, converted to STL files, and three-dimensional coordinate values were extracted. The long axes were estimated by an algorithm to synchronize the LSR line with the horizontal axis which was translated to the vertical axis. The axes of the molars defined by LSR were compared with the axes of the molars defined by PCA. The coordinate point number of each molar was 5400–5800. The algorithm for determining the tooth axes in this study consisted of four stages containing three steps each. The distance between the two axes calculated by the two methods (LSR and PCA) on the horizontal plane through the origin was less than 10^−12^ mm and the deviations between them were less than 0.003°. The long axes of the molars estimated by LSR agree almost exactly with the axes estimated by PCA, and the accuracy is sufficient for clinical usage; however, the distance between them would shorten with a more severe convergence condition of the α value at each stage of this LSR system.

## Introduction

The long axis of a tooth is one of the most important standard lines for dental analysis and treatment. In general, the long axis is defined as a two-dimensional imaginary line that passes through the cusp tip or the middle point of the crown and the apex or middle point of the root using dental or panoramic X-ray images (Wheeler 1965; Nojima et al. 2007).

The spread of cone-beam computed tomography (CBCT) has enabled precise dental analysis/diagnosis and treatment by clarification of the three-dimensional condition of the teeth, periodontal tissue, and craniofacial skeleton. For better dental treatment based on precise dental analysis/diagnosis, information about the three-dimensional axes of the teeth is as indispensable as the three-dimensional location of the teeth. The axes of the anterior teeth can be approximated with anatomical dental landmarks, such as the middle point of the incisal edge, the cusp tip of the canine crown, and the apex of the root; however, additional artificially set landmarks and reference planes are needed to determine the axes of premolars and molars because of the complicated shape of their crowns and roots.

Estimation of the axes of the molars has previously been determined through principle component analysis (PCA) with CBCT data (Kim et al. 2012; Sakamoto et al. 2015; Zhang et al. 2016). Therefore, we used the least square method. Plural approach is necessary to have the validity and reliability of the results in researches. A LSR line can be estimated by simple and popular statistical software, i.e., Microsoft Excel, without the exclusive software with high specialty. The process of estimation in this LSR method can also be visualized and confirmed. The purpose of this study was to determine the long axes of molars and their roots by ordinary least squares regression (LSR) and comparing the results with the axes estimated by PCA.

## Materials and methods

### Materials

Dry skulls kept in the Department of Anatomy at The Nippon Dental University at Niigata were used as the materials for this study. Molars with severe congenital or acquired abnormalities, defects, dental caries, heavy attrition, abnormally shaped roots, or excess resorption were excluded. Forty first molars were selected using CBCT data.

### Methods

#### Three-dimensional coordinate system with the CBCT images

**Fig. 1:**
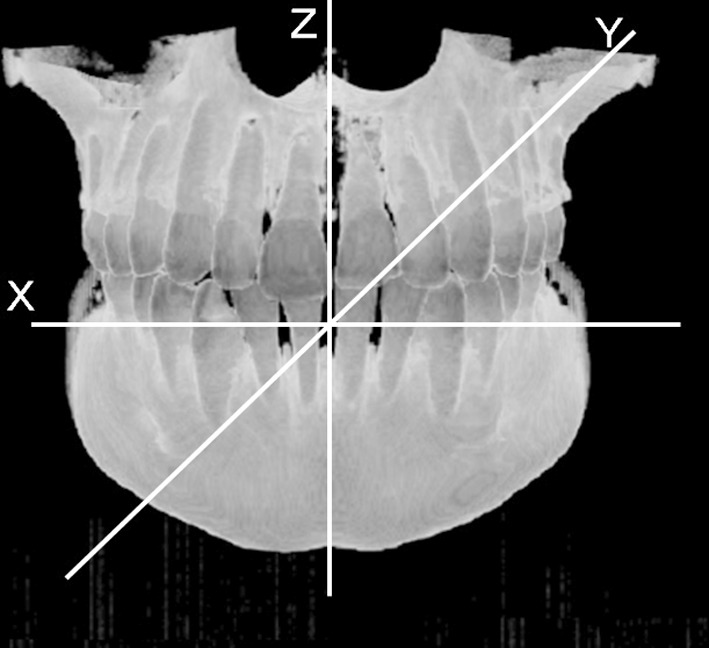
Cone-beam computed tomography image and the three-dimensional coordinate system

Three-dimensional radiological images were obtained by CBCT (3DX CT FPD8, J. Morita Corp., Suita, Japan) with a field-of-view of φ80 × 80 mm. The three-dimensional coordinate system was applied to the CBCT images (Fig. 1). The data of maxillary and mandibular molar images were extracted from the CBCT DICOM (digital imaging and communications in medicine) data with a three-dimensional image visualization system (Volume Extractor version 3.03, i-Plants Systems Ltd., Morioka, Japan). The data were reconstructed, converted and saved as standard triangulated language (STL) files with the above software. Landmarks on the surface of whole three-dimensional tooth image data were used as the coordinate values in the STL files, which could be reduced by up to 75% by simplifying the calculation of the long axes through LSR and PCA.

#### The algorithm for determining the tooth axis using LSR (Fig. 2)

Stage 0:

**Fig. 2:**
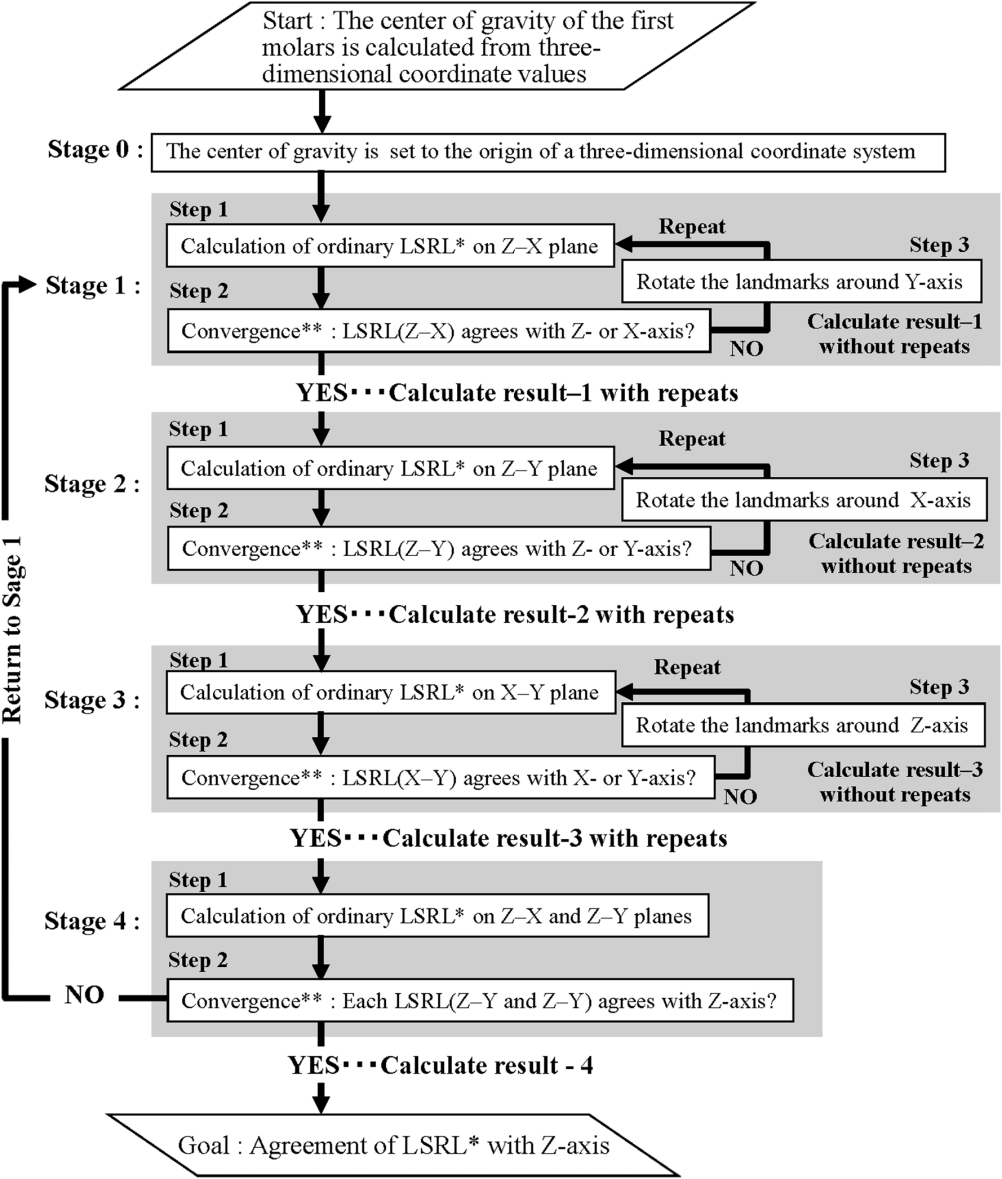
Algorithm for determining the tooth axis. *LSRL: the least square regression line. **Convergence condition: − 0.0001 < *α* < 0.0001. *α* inclination of the LSL

The center of gravity calculated from landmarks all over the tooth surface was set as the origin in the coordinate system.

Stage 1:

The least square regression line (LSRL) was calculated from the *Z* and *X* coordinate values as the explanatory and objective variables, respectively (Fig. 2 stage 1, step 1). Then, the LSRL was compared with the *Z* axis (which represented the zero value of the regression coefficient [*α*_*ZX*_]) (Fig. 2 Stage 1, Step 2). The convergence condition was set as − 0.0001 < *α*_ZX_ < 0.0001. If the LSRL did not conform to the *Z* axis, all the landmarks were rotated around the *Y* axis to make them agree (Fig. 2 Stage 1, Step 3). Calculations were repeated until the conditions were satisfied.

Stages 2 and 3:

The *Z* coordinate values and the *Y* values were set as the explanatory and objective variables, respectively in Stage 2 (Fig. 2 Stage 2, Steps 1 and 2). The coordinate values were rotated around the *X* axis (Fig. 2 Stage 2, Step 3). During this process, the *X* coordinate values and the *Y* values were set as the explanatory and objective variables, respectively (Fig. 2 Stage 3, Steps 1 and 2). The coordinate values were rotated around the Z axis (Fig. 2 Stage 2, Step 3). The convergence conditions were the same as for Stage 1. Calculations were repeated until the convergence condition was satisfied.

Stage 4:

Two LSRLs were calculated again on the *Z*–*X* and *Z*–*Y* planes (Fig. 2 Stage 4, Step 1). All regression coefficients (*α*_*ZX*_, *α*_*ZY*_, *α*_*XY*_) were checked for suitability for the convergence conditions, i.e., − 0.0001 < *α* < 0.0001. The estimated LSRL was converged by agreement with the *Z* axis within the convergence conditions (Fig. 2 Stage 4, Step 2). If it was not suitable for the convergence conditions, it was returned to Stage 1.

The calculation was performed using the LINEST function in Microsoft Excel (version 14.0.7190.5000, Microsoft).

#### Comparison between the long axes of molars calculated by PCA and LSR

The origin in the coordinate system was set to the center of gravity of the landmarks. The eigenvectors (*V*_*x*_, *V*_*y*_, *V*_*z*_) for the PCA (Sakamoto et al. 2015) were calculated with statistical software (R and R Commander, R: version 3.4.1 and Rcmdr; version 2.4-0, The R Foundation for Statistical Computing; Vienna University of Technology, Vienna, Austria). The first principal component with the highest variance was set as the tooth axis. The PCA line (PCAL) was estimated.

#### Comparison between the two long axes of molars defined by LSR and PCA

The two long axes of the molars calculated by PCA and LSR were compared. It was assumed that the line estimated by LSR agreed with the *Z* axis. The PCAL was moved with the same quantities and processes of rotations as the calculation of the LSRL. Differences between the estimated line and the *Z* axis were examined.

## Results

**Fig. 3:**
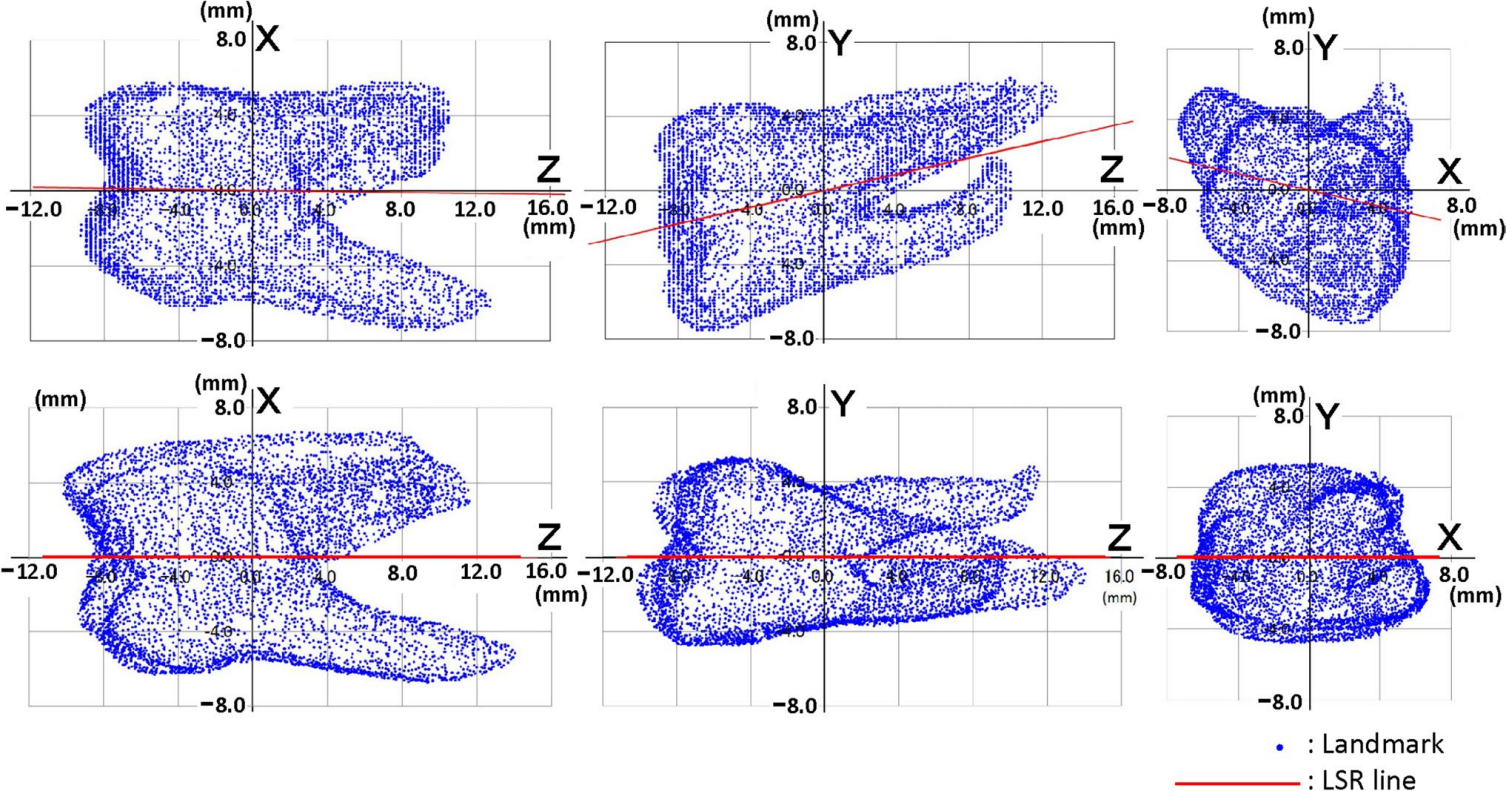
Examples of landmarks before and after calculation of the LSR. Upper: before calculation (after Stage 0) Lower: after calculation (goal). The left sides: *Z*–*X* plane (the coronal plane); the middle sides: *Z*–*Y* plane (the sagittal plane); the right sides: *X*–*Y* plane (horizontal plane)

The landmarks constructed of STL files were reduced for calculation of LSR and PCA with a three-dimensional image visualization system (i.e., 5657.4 and 5622.5 landmarks as the maxillary and mandibular first molars, respectively) (Table 1, Fig. 3). There were the upper and lower two scatter diagrams on *Z*–*X* plane (the coronal plane), *Z*–*Y* plane (the sagittal plane), and *X*–*Y* plane (horizontal plane) from the left to the right in Fig. 3.

**Table 1:** Differences between the LSR and the PCA lines of upper and lower molar

Tooth no.	Maxillary first molars				Mandibular first molars			
	Landmark no.	Distance on *X*–*Y* plane^#1^		Angulation (degree)^#2^	Landmark no.	Distance on *X*–*Y* plane^#1^		Angulation (degree)^#2^
		*X* (mm)	*Y* (mm)			*X* (mm)	*Y* (mm)	
Min	5426	2.37E − 15	2.37E − 15	1.48E − 04	5422	1.34E − 15	1.34E − 15	2.72E − 04
Max	5753	1.11E − 13	1.11E − 13	2.92E − 03	5739	5.85E − 13	5.85E − 13	2.60E − 03
Ave^#3^	5657.4	2.285E − 14	2.285E − 14	1.489E − 03	5622.5	7.366E − 14	7.366E − 14	1.080E − 03
SD^#3^	86.57	2.509E − 14	2.509E − 14	9.205E − 04	102.83	1.261E − 13	1.261E − 13	6.730E − 04

*n* = 20

^#1^Cross point of the PCA line and the *X*–*Y* plane

^#2^Angulation between the *Z* axis and the PCA line

^#3^Average and standard deviation of absolute values

As an example (Fig. 3), calculation of the LSRL of one maxillary first molar is set out below:

Stage 0 (Fig. 2 Stage 0, the upper scatter diagrams in Fig. 3):

The center of gravity calculated from all the landmarks of a maxillary first molar was moved to the origin (Fig. 2 Stage 0). Table 2 shows the three coefficients (*α*_*ZX*_, *α*_*ZY*_, *α*_*XY*_) and intercepts (*C*_*X*_, *C*_*Y*1_, *C*_*Y*2_) of the LSRLs according to the algorithm (Fig. 2).

**Table 2:** Example of a calculation of the LSRL of the maxillary first molar

Calculate result	*α*_*ZX*_^#1^	*C*_*X*_^#2^	*α*_*ZY*_^#3^	*C*_*Y*1_^#4^	*α*_*XY*_^#5^	*C*_*Y*2_^#6^
C1 without repeats	− 3.09E − 02	4.98E − 15	6.93E − 01	2.34E − 14	− 2.30E − 01	− 3.42E − 14
C1 with repeats	− 2.69E − 05	− 1.62E − 14	6.99E − 01	1.03E − 15	− 2.19E − 01	− 3.38E − 14
C2 with repeats	− 6.28E − 02	− 9.22E − 15	− 2.23E − 05	− 6.62E − 15	− 2.10E − 01	− 2.71E − 15
C3 with repeats → C4	− 5.17E − 02	− 8.69E − 15	− 4.83E − 02	− 7.20E − 15	− 3.23E − 05	− 1.20E − 14
**↓**Return to Stage 1, and recalculate until the convergence conditions are satisfied						
Re: C1 with repeats	− 3.00E − 05	− 9.93E − 15	− 4.83E − 02	− 1.05E − 14	− 8.67E − 04	− 1.21E − 14
Re: C2 with repeats	− 1.94E − 05	− 9.08E − 15	− 3.09E − 05	− 9.07E − 15	− 8.67E − 04	− 1.26E − 14
Re: C3 with repeats → C4	− 1.94E − 05	− 9.08E − 15	− 3.09E − 05	− 9.07E − 15	− 2.86E − 05	− 1.18E − 14

^#1^, ^#3^, ^#5^A coefficient of the LSRL on the *Z*–*X*, *Z*–*Y*, and *X*–*Y* plane, respectively

^#2^, ^#4^, ^#6^An intercept of the LSRL on the *X*, *Y*, and *Y* axis, respectively

Stage 1 (Fig. 2 Stage 1, Steps 1–3):

The LSRL was calculated on the *Z*–*X* plane (Fig. 2 Stage 1, Step 1) and the inclination of this line was assessed (Fig. 2 Stage 1, Step 1). If the inclination (*α*_*ZX*_) was satisfied with the convergence condition (− 0.0001 < *α*_*ZX*_ < 0.0001), the result should advance to the next step (Fig. 2 Stage 2, Table 2 C1 without repeats). If *α*_*ZX*_ was not satisfied, the landmarks were rotated around the *Y* axis (Fig. 2 Stage 1, Step 3), returning to the previous step until the convergence condition was satisfied (Fig. 2 Stage 1, Step 1).

The inclination *α*_*ZX*_ was reduced to − 2.69 × 10^−5^, and the convergence condition was satisfied (Fig. 2 calculate result-1, Table 2 C1 with repeats).

Stage 2 (Fig. 2 Stage 2, Steps 1–3):

This process was also applied to the *Z*–*Y* plane and the *X* axis (Fig. 2 Stage 2, Steps 1–3). In this stage, the inclination *α*_*ZY*_ was reduced to − 2.23 × 10^−5^, and the convergence condition of this stage was satisfied with an *α*_*ZX*_ value that deviated from the Stage 1 convergence condition, i.e., − 6.28 × 10^−2^ (Fig. 2 calculate result-2, Table 2 C2 with repeats).

Stage 3 (Fig. 2 Stage 3, Steps 1–3):

This process was applied to the *X*–*Y* plane and the *Z* axis (Fig. 2 Stage 3, Steps 1–3). The inclination *α*_*XY*_ was changed to − 3.23 × 10^−5^, and the convergence condition of this stage was satisfied with *α*_*ZX*_ and *α*_*ZY*_ values that deviated from the Stages 1 and 2 convergence conditions, i.e., − 5.17 × 10^−2^ and − 4.83 × 10^−2^, respectively (Fig. 2 calculate result-3, Table 2 C3 with repeats).

Stage 4 (Fig. 2 Stage 4, Steps 1–3):

Three LSRLs were calculated (Fig. 2 Calculate result-4, Table 2 C4). As well as the results of Stage 3 with *α* values that deviated from the convergence condition, the calculation returned to Stage 1, and recalculations (Stage 1 → 2→3 → 4) were repeated until the convergence condition was satisfied.

Re: Stage 1:

Three inclinations of the LSRLs were assessed and *α*_*ZX*_ and *α*_*ZY*_ were not satisfied with the convergence condition (Fig. 2 Stage 4, Step 2, and Table 2 C3 with repeats → C4). The calculation then returned to Stage 1 and the landmarks were rotated around the *Y* axis (Fig. 2 Stage 1, Step 3). The inclination *α*_*ZX*_ became − 3.00 × 10^−5^ (Table 2 Re: C1 with repeats).

Re: Stage 2:

The LSRLs were calculated on the *Z*–*Y* plane and assessed again (Fig. 2 Stage 2, Steps 1–3). Then the inclination *α*_*ZY*_ of this stage became − 3.09 × 10^−5^ with *α*_*ZX*_ of − 1.94 × 10^−5^ (Table 2 Re: C2 with repeats).

Re: Stage 3:

The LSRLs were calculated on the *X*–*Y* plane and assessed again (Fig. 2 Stage 3, Steps 1–3). Then the inclination *α*_*XY*_ of this stage became − 2.86 × 10^−5^ with *α*_*ZX*_ and *α*_*ZY*_ of − 1.94 × 10^−5^ and − 3.09 × 10^−5^, respectively (Table 2 Re: C3 with repeats).

Re: Stage 4:

Three LSRLs were calculated (Fig. 2 Stage 4 and Table 2 Re: C3 with repeats → C4). All inclinations of the LSRLs were assessed and satisfied with the convergence condition (Table 2 Re: C3 with repeats → C4). It was then assessed that the LSRL agreed with the *Z* axis, and the long axis of this tooth was decided as shown the lower scatter diagrams in Fig. 3.

**Fig. 4:**
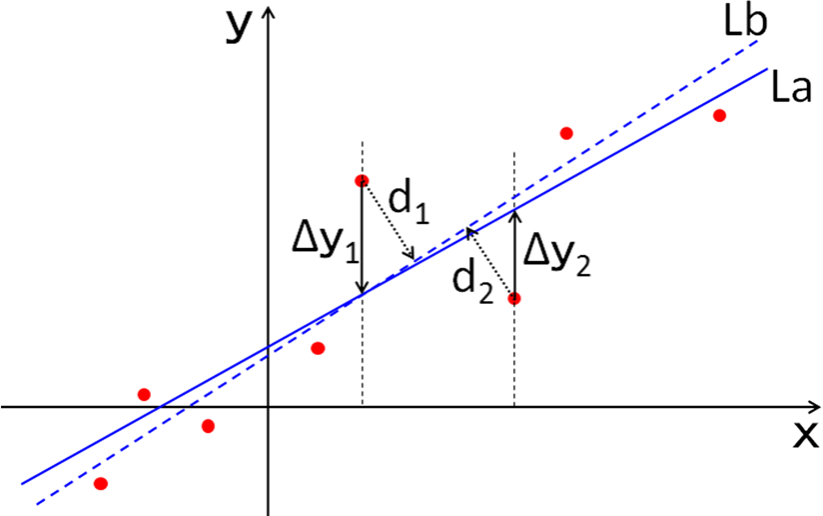
Differences between LSR and PCA. La: the line as the sum of Δ*y*_*i*(*i *= 1, 2,…*n*)_ becomes the minimum. Lb: the line as the sum of *d*_*i*(*i *= 1, 2,…*n*)_ becomes the minimum

The difference between the PCAL and the *Z* axis of each molar was also calculated. The differences between the PCAL and the *Z* axis on the *X*–*Y* plane were 2.29 × 10^−14^ mm (range 2.37 × 10^−15^ to 1.11 × 10^−13^ mm) and 2.29 × 10^−14^ mm (range 2.37 × 10^−15^ to 1.11 × 10^−13^ mm) as average values of the absolute values in the *X* and the *Y* coordinate values for the maxillary first molars respectively (Table 1, Fig. 4). Those for the mandibular first molars were 7.366 × 10^−14^ mm (range 1.34 × 10^−15^ to 5.85 × 10^−13^ mm) and 7.366 × 10^−14^ mm (range 1.34 × 10^−15^ to 5.85 × 10^−13^ mm) as average values (Table 1). The angulations between the PCAL and the *Z* axes were 1.489 × 10^−3^ (range 1.482 × 10^−4^ to 2.921 × 10^−3^) degrees and 1.080 × 10^−3^ (range 2.717 × 10^−4^ to 2.602 × 10^−3^) degrees as average values in the maxillary and mandibular first molars, respectively (Table 1).

## Discussion

Panoramic radiographs are normally used to estimate the two-dimensional long axis of teeth (Shroff 2001; Owensa and Johalb 2008; Rocha et al. 2016; Garcia-Figueroa et al. 2008). With the recent demand for high-precision dental treatment such as dental implants, extraction of impacted teeth, and orthodontic treatment, CBCT has become increasingly important in determining the three-dimensional condition of teeth and bones, including estimation of the three-dimensional tooth axis (Lee et al. 2015; Peck et al. 2007; Bouwens et al. 2011). In this study, we estimated three-dimensional tooth axes using CBCT data taken from 20 dry skulls using LSR, and found that it was possible to estimate the objective tooth axis by using the whole of the tooth surface, even in teeth with small surface defects such as tooth attrition or dental abrasion.

Previous reports about determining the three-dimensional tooth axis from CBCT data used PCA (Kim et al. 2012; Sakamoto et al. 2015; Zhang et al. 2016). Therefore, we compared the three-dimensional tooth axes defined by LSR and PCA. LSR is calculated so that the residual sum of squares becomes the minimum on the longitudinal axis (line La in Fig. 4). PCA is calculated so that the Mahalanobis distance becomes the minimum (line Lb in Fig. 4). Therefore, a difference usually exists between the LSRL and the PCAL. However, the residual values are equal to the Mahalanobis distances, as the calculated line is parallel to the horizontal axis. Then the landmarks around the first molars are rotated, until the LSRL agrees with the horizontal axis according to the algorithm, because the LSRL runs through the origin (Fig. 2).

It is impossible for the LSRL to agree with the horizontal axis mathematically, because the LSRL has an error. Then the condition of the convergence was given to assess the agreement between the LSRL and the horizontal axis. The provided condition that the absolute values of the inclination of the LSRL were smaller than 0.0001, was satisfied. The inclination of 0.0001 as the condition of the convergence was equivalent to 0.00573°. Therefore, the maximum errors produced the inclination of 0.0001 on the *X*–*Z* plane and the *Y*–*Z* plane. Accordingly, the maximum error of 0.00014 at *Z* = 1 on the *X*–*Y* plane was 0.0081°.

**Fig. 5:**
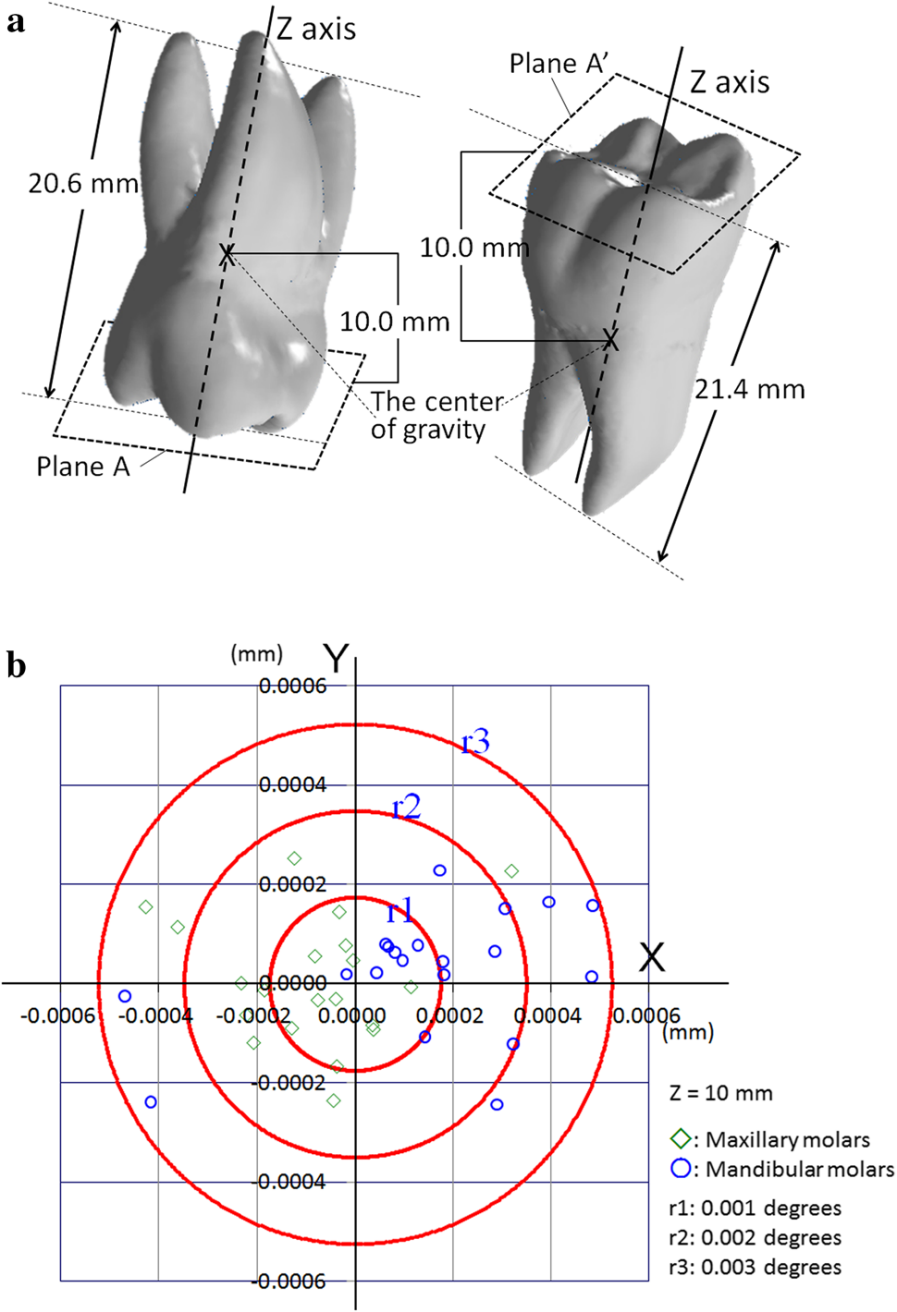
Differences between PCALs and LSRLs at *Z* = 10 mm. **a** The points of a scatter diagram corresponded to the molars. The PCAL and LSRL points are placed on the plane *A* and *A*′ set on *Z* = 10 mm from the center of gravity of molars, which are close to occlusal surfaces. **b** The scatter diagram of PCALs and LSRLS shown on the *X*–*Y* plane at *Z* = 10 mm

Some studies have reported that the length from the root to the top of the crown was about 20.7 mm and 21.1 mm in the maxillary and mandibular first molars respectively (Verhoeven et al. 1979; Viecilli et al. 2013; Hikita et al. 2018). In our study, the maximum lengths were 20.6 mm and 21.4 mm in the maxillary and mandibular first molars respectively (Fig. 5a), according to the *Z* axis. It might then be presumed that the lengths from the center of gravity to the occlusal surfaces are 10 mm. The differences between the PCAL and the LSRL were less than 0.003 mm on the *X*–*Y* plane at *Z* = 10 mm (Fig. 5b), as the LSRLs were assumed to the *Z* axis. These values may be smaller than the slice thickness of the CBCT. The convergence condition becomes more severe, as the error decreases in size. In such cases it might be useful to estimate the long axis of the maxillary and mandibular first molars with LSR.

**Fig. 6:**
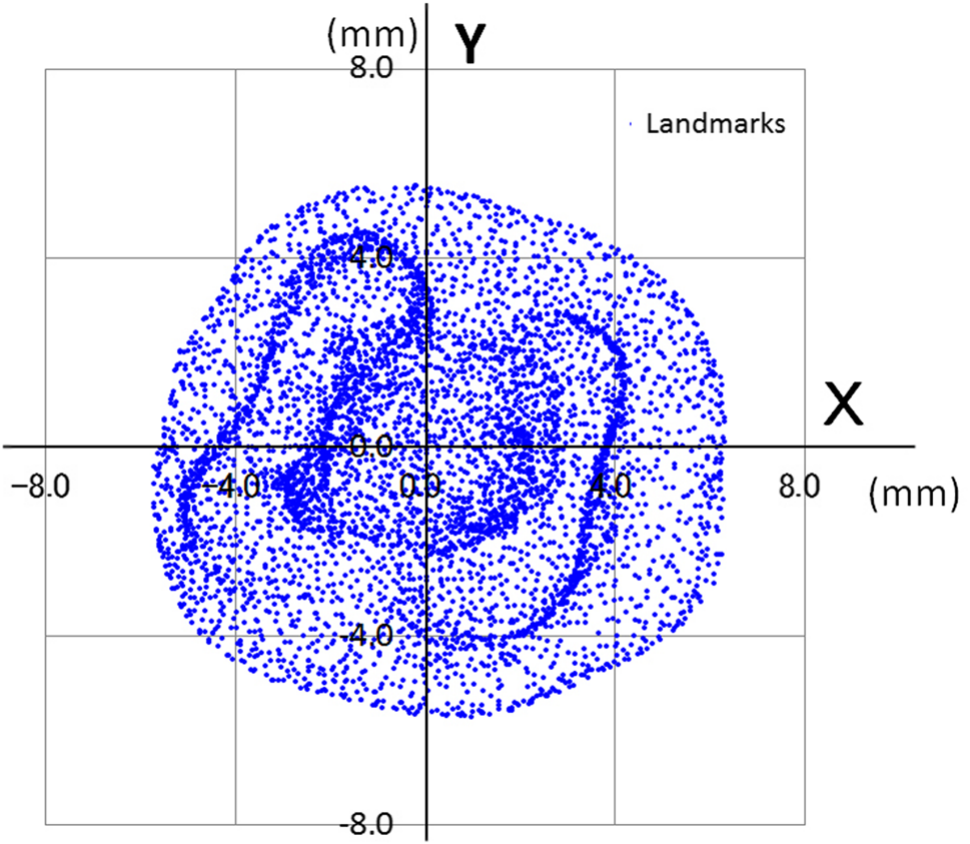
An example of the occlusal surface of the mandibular molar

The *Z* axis was estimated as the tooth axis, and the *X*–*Y* plane could be viewed as the occlusal plane. However, the first molars could not always be separated into anatomical mesial and distal parts by the *X*–*Z* plane and into the buccal and lingual parts by the *Y*–*Z* plane in the same way (Fig. 6). Therefore, a new objective process would need to be considered for separation into mesial and distal or buccal and lingual parts.

Dental treatment and research would benefit from the easy acquisition and application of digital dentistry, especially the use of three-dimensional data and its application to clinical dentistry. The individual occlusion of maxillary and mandibular molars could be assessed more precisely with knowledge of the three-dimensional tooth axes, and this information would be indispensable for simulating the occlusion with the finite element method in future research. The use of CBCT images to determine the precise positional relationship of teeth in the alveolar bone, including three-dimensional tooth axes, would help dentists to plan for better individual occlusion to prevent oral frailty, resulting in improved quality of life and increased healthy life expectancy.

## Conclusion

We developed an algorithm to synchronize the LSRLs to estimate the long axes of maxillary and mandibular first molars with multiple roots in this study. Applying this algorithm to CBCT images taken from dry skulls, the long axes of molars estimated by LSR were compared with the long axes defined with PCA.

The long axes of maxillary and mandibular first molars were estimated by using the algorithm to synchronize the LSRL with the horizontal axis, and then the inclination of the LSRLs approached zero. It was judged that the LSRLs agreed with the horizontal axis when the inclinations were over − 0.004 and under 0.004. These axes were compared with the axes defined with PCA, and the deviations between the two long axes were less than 0.003°. When we set more severe convergence conditions of the *α* value at each stage of this LSR system, the distances between the LSR and PCA tooth axes could become even smaller.

Our findings suggest that the long axes of the molars estimated by LSR agree almost exactly with the axes calculated by PCA, and the degree of accuracy is sufficient for clinical usage.
